# Accessible Low-Cost Laser Pointers for the Reduction of Aryl Halides via Triplet-Triplet Annihilation Upconversion in Aerated Gels

**DOI:** 10.3390/gels8120800

**Published:** 2022-12-06

**Authors:** Paola Domínguez Domínguez, Sebastian Bonardd, Samuel Martín Koury, Raúl Pérez-Ruiz, M. Consuelo Jiménez, David Díaz Díaz

**Affiliations:** 1 Departamento de Química Orgánica, Universidad de La Laguna, Avda. Astrofísico Francisco Sánchez 3, 38206 La Laguna, Spain; 2 Instituto de Bio-Orgánica Antonio González, Universidad de La Laguna, Avda. Astrofísico Francisco Sánchez 2, 38206 La Laguna, Spain; 3 Departamento de Química, Universitat Politècnica de València Camino de Vera, s/n, 46022 Valencia, Spain; 4 Institut für Organische Chemie, Universität Regensburg, Universitätsstr. 31, 93053 Regensburg, Germany

**Keywords:** laser pointer, photoreduction, gels, aryl halides, triplet-triplet annihilation upconversion, aerobic conditions, research economy

## Abstract

The search for economic alternatives in the use of expensive scientific equipment represents a way of providing many laboratories access to scientific developments that, otherwise, might be hampered by economic constraints. This inspired the purpose of this work, which was to demonstrate for the first time that we can carry out the photoreduction of aryl halides via green-to-blue upconversion in an aerated gel medium, using a simple economic set-up based on easily accessible and low-cost laser pointers. The optimized set-up consists of three laser pointers connected to a switching-mode power supply. One laser should be aligned to *Z*-axis and separated 5 cm from the sample, while the light incidence of the other two lasers should be adjusted to 45° and separated ca. 3 cm from the sample. The results of this study were found to be reproducible in random experiments and demonstrated that the photoreduction of several aryl halides can be carry out within 24 h of irradiation with comparable yields and mass balances, to those obtained with other very expensive pulsed laser sources. An economic estimation of the expenses concludes that we can easily reduce by >98% the total cost of this type of research by using the described set-up. Our work offers many groups with limited resources a feasible alternative to work in this area without the necessity of extremely expensive devices.

## 1. Introduction

Scientific research is nowadays very expensive in many areas, since conducting experiments requires significant human resources, consumables, and technological equipment, whose prices are gradually increasing. This is especially relevant for developing countries, with significant budget and importation constraints that limit, not only the acquisition of costly instruments, but also their maintenance over time. Obviously, many small research groups from richer countries also suffer serious difficulties to overcome the rapid obsolescence of expensive scientific equipment. Unfortunately, such limiting factors are rarely recognized by governments. Thus, the search for economic alternatives to the use of expensive scientific apparatuses is also a way of giving many laboratories access to scientific developments that are otherwise prohibited from an economic point of view.

The use of confined and compartmentalized environments constitutes one of the main strategies of nature to carry out challenging chemical reactions under mild conditions with great control in kinetic and selectivity [1,2]. This has inspired many advances in the development of biomimetic artificial nano and microreactors [3,4]. In particular, the fields of photochemistry and photocatalysis have been great beneficiaries of this approach [3,5,6,7]. Among different systems used in this context, supramolecular gels based on low molecular weight (LMW) compounds [8] self-assembled by non-covalent interactions represent highly versatile materials due to their tunable properties, easy preparation and *sol*-to-*gel* reversible transition, that can be triggered by various external stimulus (e.g., temperature, pH, etc.). The solid-like appearance and mechanical properties of gels are a consequence of the immobilization of the liquid (main component) in the interstices of the self-assembled solid matrix (secondary component) by surface tension and capillary forces. 

**Scheme 1:**
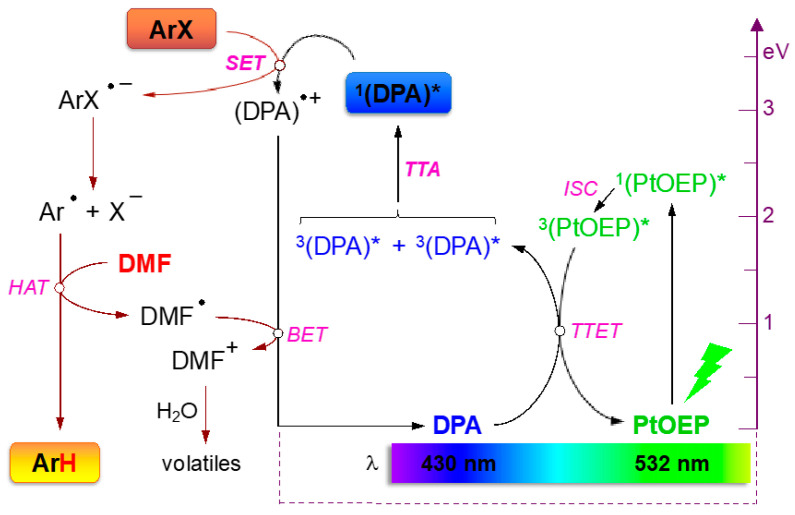
Proposed mechanism for visible light photoreduction of aryl halides at room temperature in air based on the combination of TTA-UC and SET processes in a gel. Symbol * denotes excited states. Adapted with permission from ref. [10]. Copyright 2015 Royal Society of Chemistry.

With this background, we wish to illustrate in this work the above-mentioned economical issue using, as representative example, the photoreduction of aryl halides (ArX) via photon upconversion based on triplet-triplet annihilation (TTA-UC) [9] in air using a supramolecular organogel as reaction medium [10]. From a mechanistic point of view, the intragel reaction involves a sequential TTA, single electron transfer (SET) and H-atom transfer (HAT). As shown in Scheme 1, the process begins by the selective excitation of a sensitizer (i.e., platinum (II) octethyl-porphyrin (**PtOEP**)) at 532 nm to generate the corresponding excited triplet state, ^3^(**PtOEP**)*, after efficient intersystem crossing (ISC). Subsequently, **PtOEP** could be restored upon triplet-triplet energy transfer (TTET) with 9,10-diphenylanthracene (**DPA**) (emitter), giving rise to long-lived excited triplet state of **DPA**, ^3^(**DPA**)*. Subsequently, a collision between two ^3^(**DPA**)* molecules effects population of the ^1^(**DPA**)* state. These species can induce SET to the electrophilic aryl halides, leading to the formation of the unstable radical anions ArX˙^−^, which undergo fragmentation affording the corresponding aryl radicals Ar˙ and anions X^−^. Finally, rapid HAT from solvent (DMF) molecules yields the formal reduction product ArH. The formed radical DMF˙ may regenerate **DPA** by back electron transfer (BET) giving highly electrophilic DMF^+^ species, which are known to be hydrolyzed into volatile products upon work-up [10]. 

This technology based on TTA-UC [11] has received considerable attention during the past decade due to its low excitation power, tunable excitation and emission wavelengths, and multiple applications in different areas. This technology enables, for instance, the transformation of visible light into UV light involving a bimolecular system (i.e., a sensitizer or donor and an emitter or acceptor) [12,13,14,15]. In 2015, we managed to carried out the first intragel photoreduction of aryl halides in modest yields via a TTA-UC mechanism using a high-intensity pulsed Nd:YAG laser instrument, while LED irradiation afforded much lower conversions (e.g., <20%) [10]. Unfortunately, the present price for such pulsed laser equipment is around several thousand euros, which makes them scarce devices in many universities and research groups. In this context, and due to the poor results with LED-based set ups, we were motivated by the possibility of making these experiments more economically-accessible using a proper set up of low-cost high-intensity laser pointers. This type of pointers can be purchased in the marked by less than a hundred euros.

## 2. Results and Discussion

The TTA-UC process is nowadays one of the most powerful wavelength conversion technologies [16,17]. The mechanism involves the initial absorption of low-energy photons by the donor, generating its singlet excited state that evolves to the triplet excited state through intersystem crossing. Then, triplets of the acceptor are populated by triplet–triplet energy transfer from the triplets of the sensitizer via Dexter mechanism. When two acceptor molecules in their triplet states undergo a collision, a higher singlet energy level is formed by TTA and, consequently, generates a delayed upconverted fluorescence. Unfortunately, TTA-UC processes in solution require anaerobic conditions to prevent triplet quenching by oxygen [18]. In order to block oxygen diffusion, the TTA-UC process has been performed with different degree of success under aerated conditions using, for instance, different solid polymers [19], viscous liquids [20] or viscoelastic gels [21] as support media. In a previous work, we demonstrated the TTA-triggered intragel photoreduction of aryl halides [10]. Therein, the gel network provided a stable, confined, and compartmentalized microenvironment for the challenging multistep process under aerobic conditions, visible light irradiation, at room temperature, ambient pressure and without additional additives [21,22,23]. In addition, the use of a pulsed laser ensured a high concentration of acceptor triplets in the gel medium and, hence, maximize their probability of collision. 

**Figure 1:**
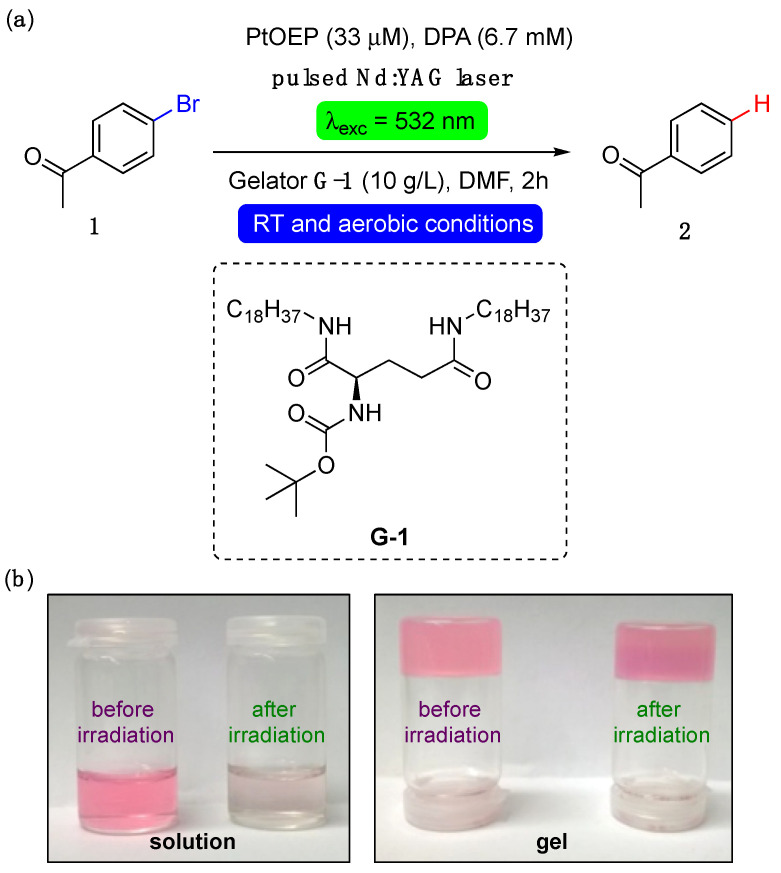
(**a**) Scheme of photoreduction of 4′-bromoacetophenone (**1**) promoted by TTA-UC and performed in a supramolecular gel made of LMW gelator **G-1**. (**b**) Photographs of doped solutions before and after irradiation showing decolorization caused by degradation of **PtOEP** (*left*) and photographs of doped gel before and after irradiation (*right*). Adapted with permission from ref. [10]. Copyright 2015 Royal Society of Chemistry.

We have achieved the intragel photoreduction of 4′-bromoacetophenone (**1**) to acetophenone (**2**) in 58% yield via TTA-UC [10]. For this, we used *N*,*N’*-dimethylformamide (DMF) as solvent, the well-known pair **PtOEP** (sensitizer)—**DPA** (emitter) [24,25], *N*,*N’*-bis(octadecyl)-l-boc-glutamic diamide (**G-1**) as LMW gelator [26] and 2 h of irradiation with a 532 nm high energy pulsed laser (Figure 1A). When the reaction was performed in aerated DMF, complete decolorization of the original pink solution was observed after irradiation due to the decomposition of PtOEP by diffused molecular oxygen. In contrast, there was no change in the pink color in gel phase, which proved an efficient confinement effect of the gel network, preventing a rapid quenching of the excited species by dissolved oxygen (Figure 1B). Importantly, the result obtained in aerobic gel phase was comparable to that obtained in solution under strict inert atmosphere. Furthermore, rheological measurements of the gel matrix before and after laser irradiation also demonstrated the preservation of the gel integrity in terms of thermal and mechanical properties [10]. 

Taking advantage of the great oxygen-blocking effect of the gel medium, we hypothesized that high intensity laser pointers, placed in a proper set up, could afford the formation of enough long-lived acceptor triplets over time to induce the TTA-UC cascade. This would allow us to perform these transformations in a standard synthesis lab, with rapidly accessible low-cost laser pointers instead of delicate and costly pulsed lasers. To demonstrate that specific point constitutes the main objective and focus of the present study.

### 2.1. Design of the Set-Up Using Laser Pointers

For the initial optimization experiments, the photoreduction reaction of **1** was studied in DMF using the same concentrations of reactants as those previously optimized and employed with the pulsed laser [10,21], i.e., [**1**] = 10 mM, [**PtOEP**] = 33 μM, [**DPA**] = 6.7 mM, [**G-1**] = 10 g/L, [dodecane] = 10 mM (internal standard). Preliminary experiments demonstrated that those values also remained optimal in the case of laser pointers (Tables S1 and S2). Yields were estimated by GC-FID measurements, with an intra- and inter-batch variability of ±3%. It should be emphasized that in this study we have used the same model reaction and substrates employed in our previous contribution [10]. Moreover, the reaction products are commercially available, which enables the correct identification and quantification of the compounds by GG-FID using a proper calibration curve (Figure S1) as we have previously demonstrated [10].

#### Initial Set-Up and Optimization Studies

**Figure 2:**
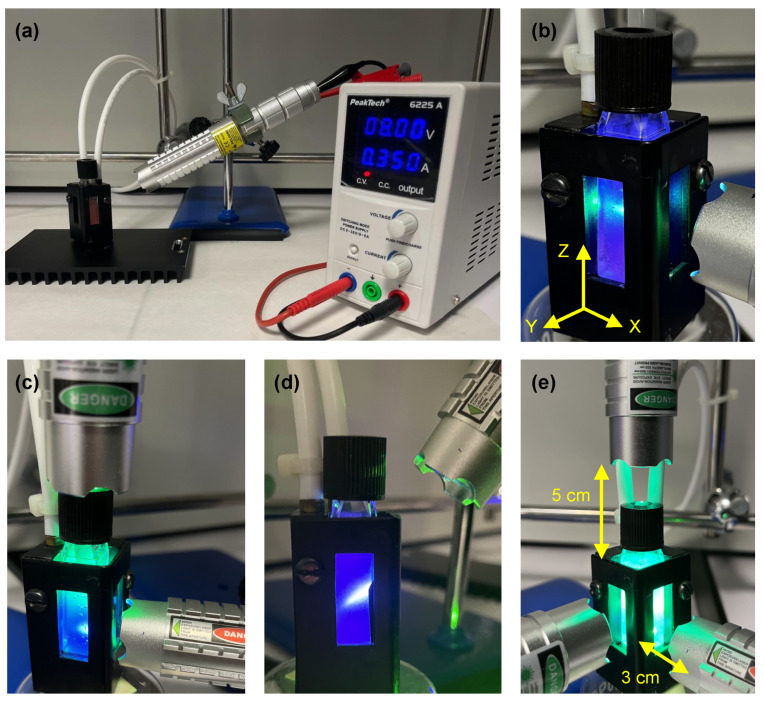
(**a**) Irradiation set-up used in this work for the intragel photoreduction of aryl halides by TTA-UC using low-cost laser pointers. Representative pictures of green-to-blue upconversion under aerobic conditions achieved with: (**b**) 1 laser pointer aligned to *X*-axis; (**c**) 2 perpendicular laser pointers; (**d**) 1 laser pointer with light incidence adjusted at 45°; and (**e**) 3 laser pointers aligned to the xyz-coordinated axes. General reaction conditions: [**1**] = 10 mM, [**PtOEP**] = 33 μM, [**DPA**] = 6.7 mM, [**G-1**] = 10 g/L, λ_exc_ = 532 nm, DMF = 3 mL.

Figure 2a shows the general set-up consisting of a laser pointer connected to a switching-mode power supply, with the voltage and amperage adjusted to 0.8 V and 0.35 A, respectively. The cuvette containing the corresponding gel was placed into a water-jacketed cell holder to minimize any thermal effect. 

Firstly, we demonstrated that the simultaneous use of two perpendicular laser pointers (i.e., one in the axial direction and the other one in the transverse direction), instead of only one, afforded the best results when the distances between the laser pointers and the cuvette were adjusted to 5 cm in the axial direction and 3 cm in the transverse direction (Figure 2b,c; Table 1, entry 4 vs. entries 1–3). It is worth mentioning that the use of other distances varying between 1 and 7 cm in any of the mentioned directions caused a drop in the reaction yields (Table S3). 

In terms of irradiation time, the highest yields were achieved after 24 h. The use of longer irradiation times (e.g., 48 h) afforded only minor increments in the final yield (ca. 5%) (Table S4). On the other hand, the use of 2 h afforded only traces (<3%) of the desired product. Therefore, we decided to fix the reaction time for this study in 24 h. Although the use of a pulsed laser reduced the reaction time at 2 h [10], the longer reaction times required with the pointers set up was well-compensated by nearly quantitative mass balances as well as great economical and accessibility advantages of the proposed system. 

Furthermore, we found that not only the number of pointers was relevant, but also the angle of incidence of the light beam was critical. The best results were found when the laser pointers, initially parallel to XY-plane, were reoriented to achieve the light incidence at 45°, which would increase the path travelled by the light through the gel sample (Figure 2d; Table 1, entry 5 vs. entry 1). Therefore, a larger area of the gel sample would be exposed to the photons from the laser beam using an incidence at 45°, thus increasing the chance for the collision of triplets. Finally, we demonstrated that the simultaneous use of three perpendicular laser pointers greatly improved the reaction yield until ca. 50.1% (Figure 2e; Table 1, entry 6). This value was comparable to that obtained with the pulsed laser [10].

### 2.2. Substrate Scope under Optimized Conditions

Table 2 outlines the results of the photoreduction of different aryl halides with the optimized conditions and the set up using three laser pointers (i.e., one laser aligned to *Z*-axis and two lasers with the light incidence adjusted to 45° in each irradiation window of the cell holder). As we previously demonstrated [10], substituted aryl bromides with very high reduction potentials (i.e., alkyl-, MeO-, CF_3_-, NO_2_-substituted compounds) show no photoreduction activity under green laser irradiation, which is in good agreement with the free energy changes, estimated using the Rehm-Weller equation, associated with the electron transfer taking place from the **DPA** excited singlet state to the substrate. However, other substrates were photo-reduced using the laser pointers set-up affording the desired products in yields comparable to those obtained with the pulsed laser [10]. It should be emphasized that the observed differences in reactivity can be correlated with the energy activation barriers previously calculated for these compounds [10,27]. It is also very important to emphasize that the results obtained with the described set up were reproducible through multiple experiments randomly assigned to different researchers involved in the project. 

### 2.3. Room for Future Improvement: Dinamic Trajectory of the Laser Pointer

**Figure 3:**
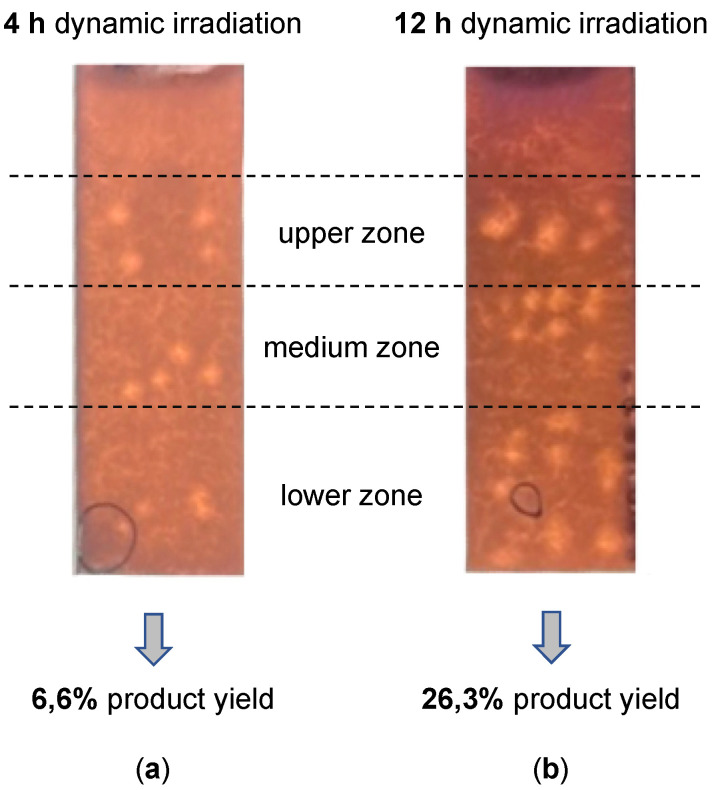
Doped gel after dynamic irradiation along the sample with a laser pointer during (**a**) 4 h and (**b**) 12 h. Conditions: [**1**] = 10 mM, [**PtOEP**] = 33 μM, [**DPA**] = 6.7 mM, [**G-1**] = 10 g/L, [dodecane] = 10 mM (int. std.), λ_exc_ = 532 nm, DMF = 3 mL. Product yields were calculated by GC-FID.

As mentioned in the previous section, the reaction yield is greatly improved when the laser pointer irradiates the gel sample with an orientation of ca. 45° with respect to *Z*-axis. This increase can be related to a larger trajectory of the light beam through the gel sample. With this in mind, we decided to carry out an experiment applying a dynamic trajectory to the laser pointer. For this, we manually changed the trajectory of the laser every 20 min along the gel sample and within the irradiation window, distributing the focus point equally in three zones of the cuvette (i.e., upper, medium and lower zones). For comparative purposes this preliminary test was carried out using only one laser pointer oriented parallel to XY-plane. The focus points of the laser beam were easily visualized in the gel sample as pale pink spots that were generated during the irradiation with the laser focused on that exact location (Figure 3). When the laser pointer was manually moved to a different location, a new bright spot was observed on the gel surface. In other words, the pale spots provide a visual representation of the exact locations where the laser light beam reached the sample. It is worth mentioning that no macroscopic *sol*-to-*gel* transition was observed during the irradiation experiments, not even at the bright spots.

As shown earlier, static irradiation of a gel sample afforded the desired product in 7.8% yield within 24 h (Table 1, entry 1). However, changing the position of the pointer every 20 min enabled the formation of the desired product with a yield of 6.6% within 4 h and 26,3% within 12 h (Figure 3).

Therefore, the application of dynamic trajectories of the laser beams in the discussed set-up might help for a further increase in the product yield. Although providing dynamic trajectories to the laser pointers represents at this point a technical challenge, especially if an automatized operation is desired, these last results indicate that there is still room for improvement in our set up to carry out reliable research in this area while minimizing economic constraints.

## 3. Conclusions

In conclusion, the use of high intensity low-cost laser pointers enables to carry out, for instance, the photoreduction of aryl halides via green-to-blue upconversion (i.e., TTA-UC) in an aerated gel medium. In this particular case, we have described the set up consisting of three laser pointers connected to a switching-mode power supply. One laser should be aligned to *Z*-axis and separated 5 cm from the sample, while the light incidence of the other two lasers should be adjusted to 45° in each irradiation window of a water-jacketed cell holder (distance between those lasers and the sample should be ca. 3 cm). Photoreduction of several aryl halides required 24 h of irradiation, a significant longer time compared to the use of a pulsed laser source to get comparable yields. However, our set-up represents a remarkable economic and reproducible alternative to the use of very expensive pulsed laser, providing global access to this type of research. Furthermore, the application of dynamic trajectories of the laser beams represents a room for improvement in future development. An economic estimation of the expenses indicates that we can easily reduce by >98% the total cost of this type of research by using the described set-up. 

## 4. Materials and Methods

### 4.1. Materials

4′-Bromoacetophenone (CAS 99-90-1, 98%, Sigma-Aldrich, Burlington, MA, USA); 2′-bromoacetophenone (CAS 2142-69-0, 99%, Aldrich); 4′-iodoacephenone (CAS 13329-40-3, ≥97%, Sigma-Aldrich); 2-bromoquinoline (CAS 2005-43-8, 95%, Sigma-Aldrich); 3-bromoquinoline (CAS 5332-24-1, 98%, Sigma-Aldrich); 4-iodoaniline (CAS 540-37-4, 98%, Sigma-Aldrich); 9,10-diphenylanthracene (**DPA**) (CAS 1499-10-1, 97%, TCI Europe); platinum octaethylporphyrin (**PtOEP**) (CAS 31248-39-2, 95%, Sigma-Aldrich); dichloromethane (DCM) (CAS 51325-91-8, 98%, Sigma-Aldrich), *N*,*N’*-dimethylformamide (DMF) (CAS 68-12-2, p.a. grade, Sigma-Aldrich), dimethyl sulfoxide (DMSO) (CAS 67-68-5, p.a. grade, Sigma-Aldrich) and anhydrous dodecane (CAS 112-40-3, >99%, Sigma-Aldrich) were used as received without further purification. Compound *N*,*N’*-bis(octadecyl)-L-Boc-glutamic diamide (**G1**) was synthesized following the general procedure previously reported [28,29]. Briefly, *N*-Boc-L-glutamic acid (0.01 mol, 1.0 equiv) and octadecylamine (0.02 mol, 2.0 equiv) in dichloromethane (200 mL) were mixed. Then, 1-ethyl-3-(3-(dimethylamino)propyl)carbodiimide hydrochloride (EDC·HCl) (0.022 mol, 2.2 equiv) was added to the mixture and stirred at RT for 72 h. The obtained white solid was isolated by filtration and washed with DCM (×3). The crude product was dissolved in THF and precipitated by water, affording the desired product was a white solid (80% yield): ^1^H NMR (300 MHz, CDCl_3_), δ (ppm): 0.87 (t, 6H, *J* = 6.8 Hz, CH_3_); 1.25 (br s, 60H, CH_2_), 1.43 (s, 9H, CH_3_); 1.46–1.58 (m, 4H, CH_2_); 1.93–2.06 (m, 2H, CH_2_); 2.27 (m, 2H, CH_2_); 3.22–3.26 (m, 4H, CH_2_), 4.08 (br s, 1H, CH); 5.77 (br s, 1H, NH); 6.32 (br s, 1H, NH); 6.69 (br s, 1H, NH).

Laser pointers (<500 mW, 532 nm) were purchased from TorLaser and Comprarlaser. PeakTech^®^ 6225 A switching power suppliers (0–30 V/0–5 A; resolution 10 mV/1 mA) were connected to the laser pointers (see Figure S1 for more details) to enable continuous irradiation over several hours. Screw capped Hellma^®^ fluorescence cuvettes, standard cells, Macro, were purchased from Sigma-Aldrich (Suprasil^®^ quartz, spectral range 200–2500 nm, pathlength 10 × 10 mm, chamber volume 3500 μL).

### 4.2. Methods

#### 4.2.1. General Procedure for the Preparation of Doped Gels

A weighed amount of all required components (i.e., LMW gelator, substrate, internal standard and TTA-UC pair) and the appropriate solvent were placed in a screw-capped quartz cuvette and gently heated with a heat gun until an isotropic solution was obtained. Subsequently, this resulting homogeneous solution was allowed to cool down until the formation of the gel-like system was observed in a uniform manner. No control over the temperature rate was applied during the heating-cooling process. Gel formation was initially established when the materials did not exhibit gravitational flow upon turning the cuvette upside-down. The viscoelastic nature of the gel state was previously confirmed by oscillatory rheological measurements [5]. Note: the quantities used of each component are defined in the main text and supporting information. 

#### 4.2.2. General Procedure for Steady-State Irradiations

The cuvette containing the corresponding doped gel was placed into a water-jacketed cell holder having two perpendicular irradiation windows. The sample was irradiated under aerobic conditions and specific parameters (see below) with a low-cost commercial laser pointer of 532 nm as selective wavelength, which was connected to a power supply (voltage and amperage were adjusted at 8.0 V and 0.35 A, respectively, according to the specifications of the laser pointer (<500 mW; see Supporting Information for details concerning the building of the laser set-up). After irradiation, dichloromethane (3 mL) was added and washed with brine (1.5 mL). The organic phase was separated, dried (anhydrous Na_2_SO_4_) and filtered for further analysis. Reaction conversions and total yields were quantified by GC-FID with a proper internal standard (see Section 4.2.3). As defined in the main text and supporting information, we performed irradiation experiments involving different special orientations of laser pointers, number of laser pointers, distance between the pointer and the cuvette, a new dynamic pointer trajectory, as well as changes in the concentration of the gelator and the TTA-UC pair. Experiments were randomly done at least in duplicate. Estimated standard deviation of the yield was found to be ±3%.

#### 4.2.3. GC-FID Quantification

GC-FID spectra were obtained on an Agilent 8860 GC-FID instrument. Note that GC techniques offers low detection limits for molecules with a molecular weight below 1250 Da. Calibration was carried out using a 5-point calibration against a 10 mM dodecane internal standard. The oven temperature program was set up as following: Initial temperature (50 °C) was kept for 0.5 min. Then, it was increased at a rate of 25 °C/min until the final temperature (280 °C) was reached and kept for 10 min. Calibration curve and a representative GC-FID chromatograms are shown in Figures S2 and S3, respectively. Products were previously characterized by NMR confirming that isolated yields were in good agreement with those calculated by GC analysis [10] (Figure S4).

## Figures and Tables

**Table 1:** Photoreduction of 4′-bromoacetophenone (**1**) using laser pointers with different configurations ^1^.

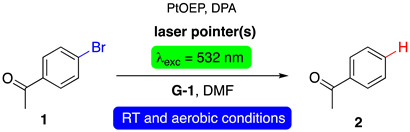		
Entry	Number of Laser Pointers (Relative Position and Angle of Incidence ^2^)	Yield 2 (%) ^3^
1	1 (lateral side at 0°)	7.8
2	1 (top side at 90°)	10.6
3	2 (both lateral sides at 0°)	12.3
4	2 (top side at 90° + one lateral side at 0°)	36.9
5	1 (lateral side at 45°)	28.8
6	3 (top side at 90° + both lateral sides at 45°)	50.1

^1^ General conditions: [**1**] = 10 mM, [**PtOEP**] = 33 μM, [**DPA**] = 6.7 mM, [**G-1**] = 10 g/L, [dodecane] = 10 mM (int. std.), 24 h, λ_exc_ = 532 nm, DMF = 3 mL. ^2^ The distances between the laser pointers and the cuvette were 3 cm (lateral sides) and 5 cm (top side). The light incidence angle with respect to the XY-plane is indicated in each case. ^3^ Values calculated by GC-FID. Standard deviation ±3%.

**Table 2:** Photoreduction of aryl halides using three laser pointers with optimal configuration ^1^.

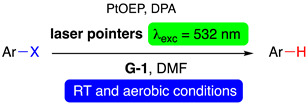			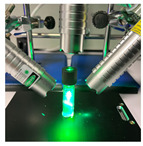
Entry	Aryl Halide Ar-X	Aryl Product Ar-H	Product Yield (%) ^2^
1	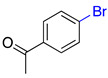	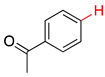	50,1
2	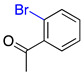	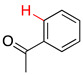	49,6
3	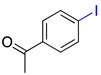	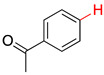	33,5
4	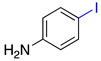	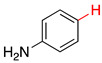	7,2
5	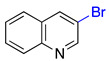	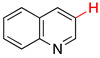	10,1
6	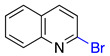	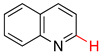	9,2

^1^ General conditions: [**Ar-X**] = 10 mM, [**PtOEP**] = 33 μM, [**DPA**] = 6.7 mM, [**G-1**] = 10 g/L, [dodecane] = 10 mM (int. std.), 24 h, λ_exc_ = 532 nm, DMF = 3 mL. Three laser pointers were used with the configuration shown in the inset picture. Distances between the laser pointers and the cuvette were 3 cm (lateral sides, 45° incidence) and 5 cm (top side). ^2^ Values calculated by GC-FID. Standard deviation ±3%.

## Data Availability

The data generated from the study are presented and discussed in the manuscript and Supplementary Materials.

## Supplementary Materials

The following supporting information can be downloaded at: https://www.mdpi.com/article/10.3390/gels8120800/s1, Table S1: Photoreduction of 4′-bromoacetophenone (**1**) using two perpendicular laser pointers and different concentrations of the TTA-UC pair; Table S2: Photoreduction of 4′-bromoacetophenone (**1**) using two perpendicular laser pointers and different gelator concentrations; Table S3: Photoreduction of 4′-bromoacetophenone (**1**) using two perpendicular laser pointers located at different distances from the sample; Table S4: Photoreduction of 4′-bromoacetophenone (**1**) using two perpendicular laser pointers and different irradiation times; Figure S1: Calibration curve of acetophenone by GC-FID; Figure S2: Representative GC-FID chromatogram of the photoreduction of 4′-bromoacetophenone (**1**) using laser pointers; Figure S3: Representative GC-FID chromatograms of the photoreduction of different substates using laser pointers. The chromatograms of commercial reactants and products are also shown in each case as reference; Figure S4: Representative ^1^H NMR spectra of reaction products.
