# Supplementary material for: Accessible Low-Cost Laser Pointers for the Reduction of Aryl Halides via Triplet-Triplet Annihilation Upconversion in Aerated Gels
Source: Gels. 2022 Dec 6;8(12):800. doi: 10.3390/gels8120800 (PMC9778448; doi:10.3390/gels8120800)
Supplement: Supplementary file 1 [file gels-08-00800-s001.zip › gels-2027347-supplementary.pdf]

# *Supporting Information*

## Accessible low-cost laser pointers for the reduction of aryl halides via triplet-triplet annihilation upconversion in aerated gels

Paola Domínguez Domínguez, Sebastian Bonardd, Samuel Martín Koury, Raúl Pérez-Ruiz, M. Consuelo Jiménez, and David Díaz Díaz*

**Table of contents**


| Table/Figure | Caption | Page |
|---|---|---|
| Table S1 | Photoreduction of 4'-bromoacetophenone (**1**) using two perpendicular laser pointers and different concentrations of the TTA-UC pair | S3 |
| Table S2 | Photoreduction of 4'-bromoacetophenone (**1**) using two perpendicular laser pointers and different gelator concentrations | S3 |
| Table S3 | Photoreduction of 4'-bromoacetophenone (**1**) using two perpendicular laser pointers located at different distances from the sample | S3 |
| Table S4 | Photoreduction of 4'-bromoacetophenone (**1**) using two perpendicular laser pointers and different irradiation times | S4 |
| Figure S1 | Connection setup: The red cable is placed on the negative pole of the power supply and the black cable on the positive pole of the power supply (1). Subsequently, the special hook of the red cable is connected to the spring that we find inside the laser and the cable with a crocodile head is placed on the side of the laser (2), leaving our system completely connected (3) | S4 |
| Figure S2 | Calibration curve of acetophenone by GC-FID | S5 |
| Figure S3 | Representative GC-FID chromatograms of the photoreduction of different substates using laser pointers. The chromatograms of commercial reactants and products are also shown in each case as reference | S6-S8 |
| Figure S4 | Representative $^{1}$H NMR spectra of reaction products | S9 |

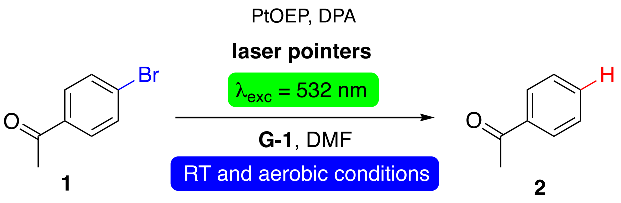


**Table S1.** Photoreduction of 4'-bromoacetophenone (**1**) using two perpendicular laser pointers and different concentrations of the TTA-UC pair. [1]

| Entry | [PtOEP]/[DPA] | Yield **2** (%) [2] |
|---|---|---|
| 1 | 33.0 µM/6.7 mM | 36.9 |
| 2 | 68.6 µM/5.0 mM | 29.5 |
| 3 | 99.0 µM/9.9 mM | 21.7 |

[1] General conditions: [**1**] = 10 mM, [**G-1**] = 10 g/L, [dodecane] = 10 mM (int. std.), 24 h, $\lambda_{exc}$ = 532 nm, DMF = 3 mL. The distances between the laser pointers and the cuvette were 3 cm (lateral side) and 5 cm (top side). [2] Standard deviation ± 3%.

**Table S2.** Photoreduction of 4'-bromoacetophenone (**1**) using two perpendicular laser pointers and different gelator concentrations. [1]

| Entry | [**G-1**] | Yield **2** (%) [2] |
|---|---|---|
| 1 | 10 g/L | 36.9 |
| 2 | 20 g/L | 25.2 |

[1] General conditions: [**1**] = 10 mM, [PtOEP]= 33 µM, [DPA] = 6.7 mM, [dodecane] = 10 mM (int. std.), 24 h, $\lambda_{exc}$ = 532 nm, DMF = 3 mL. The distances between the laser pointers and the cuvette were 3 cm (lateral side) and 5 cm (top side). [2] Standard deviation ± 3%.

**Table S3.** Photoreduction of 4'-bromoacetophenone (**1**) using two perpendicular laser pointers located at different distances from the sample. [1]

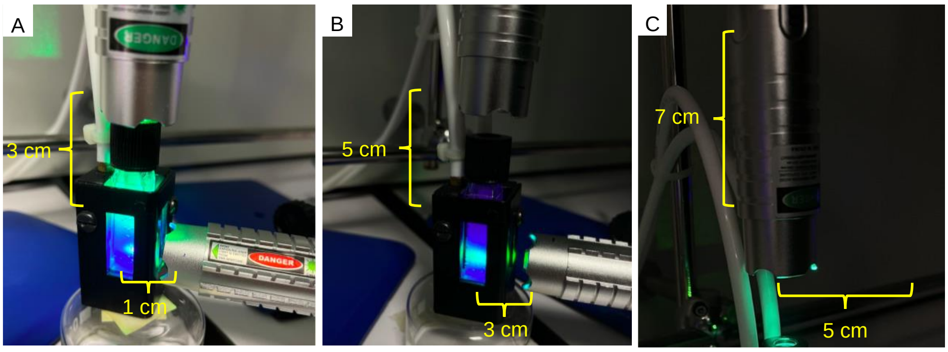


| Configuration | Yield **2** (%) [2] |
|---|---|
| A | 26.3 |
| B | 36.9 |
| C | 31.4 |

[1] General conditions: [**1**] = 10 mM, [**G-1**] = 10 g/L, [PtOEP]= 33 µM, [DPA] = 6.7 mM, [dodecane] = 10 mM (int. std.), 24 h, $\lambda_{exc}$ = 532 nm, DMF = 3 mL. [2] Standard deviation ± 3%.

**Table S4.** Photoreduction of 4′-bromoacetophenone (**1**) using two perpendicular laser pointers and different irradiation times.[1]

| Entry | Time | Yield **2** (%)[2] |
|---|---|---|
| 1 | 4 h | 8.0 |
| 2 | 12 h | 15.4 |
| 3 | 24 h | 36.9 |
| 4 | 48 h | 41.4 |

[1] General conditions: [**1**] = 10 mM, [**G-1**] = 10 g/L, [PtOEP]= 33 μM, [DPA] = 6.7 mM, [dodecane] = 10 mM (int. std.), 24 h, $\lambda_{exc}$ = 532 nm, DMF = 3 mL. The distances between the laser pointers and the cuvette were 3 cm (lateral side) and 5 cm (top side). [2] Standard deviation ± 3%.

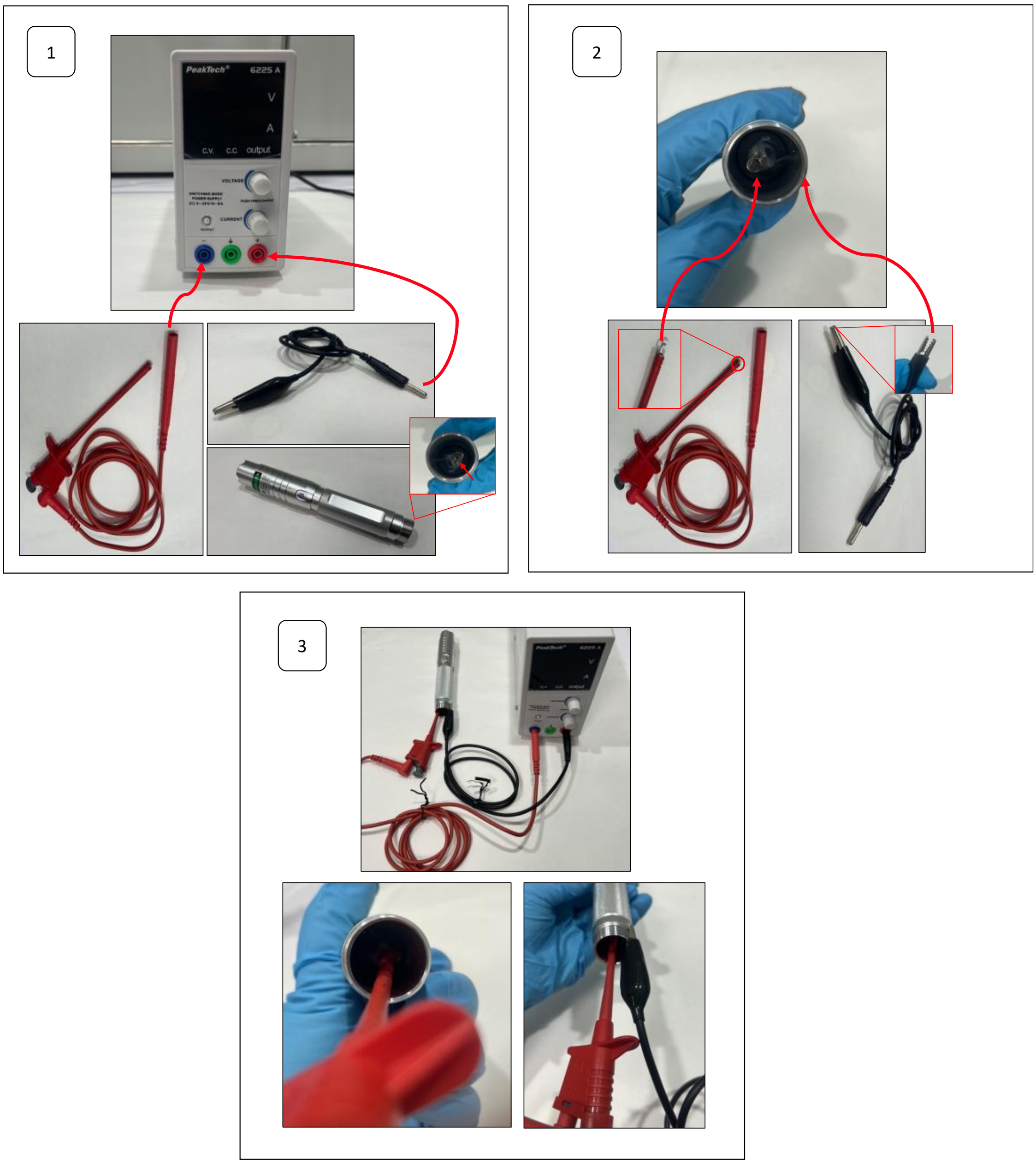


**Figure S1.** Connection setup: The red cable is placed on the negative pole of the power supply and the black cable on the positive pole of the power supply (1). Subsequently, the special hook of the red cable is

connected to the spring that we find inside the laser and the cable with a crocodile head is placed on the side of the laser (2), leaving our system completely connected (3).

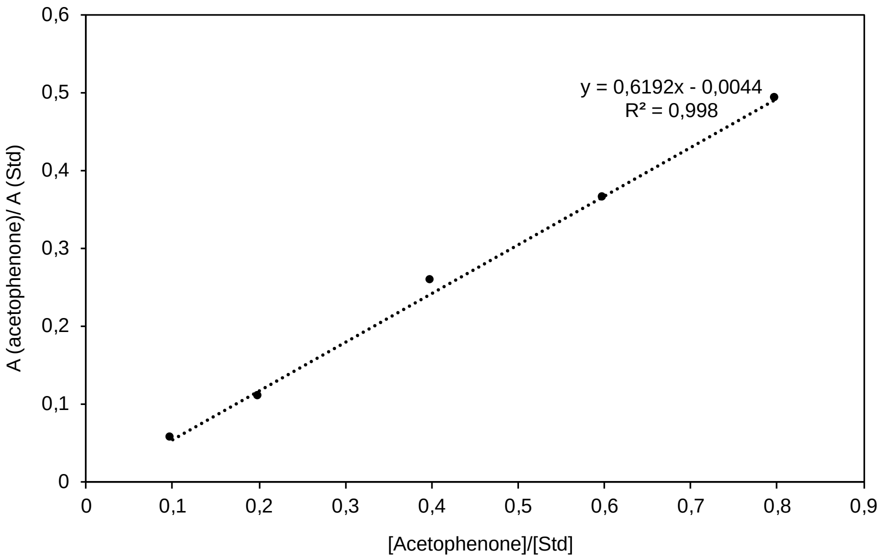


**Figure S2.** Calibration curve of acetophenone by GC-FID.

**Chromatogram of comercial reactant 2-bromoacetophenone**

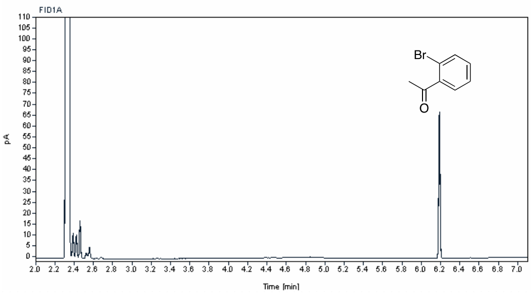


**Chromatogram of the commercial product acetophenone**

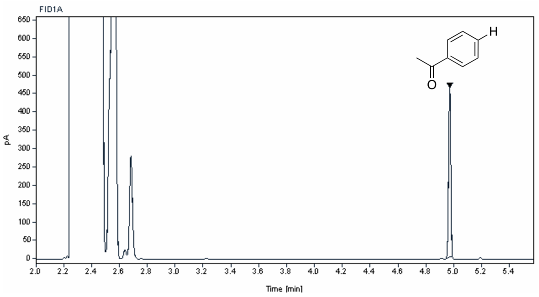


**Chromatogram after reaction**

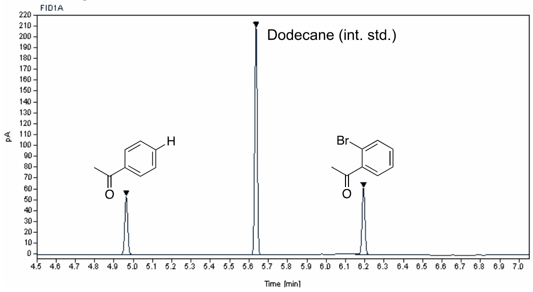


**Chromatogram of comercial reactant 4'-iodoacetophenone**

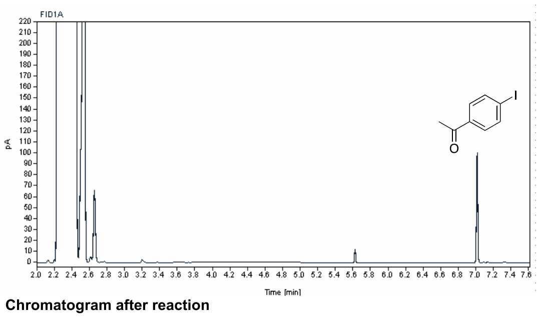


**Chromatogram of the commercial product acetophenone**

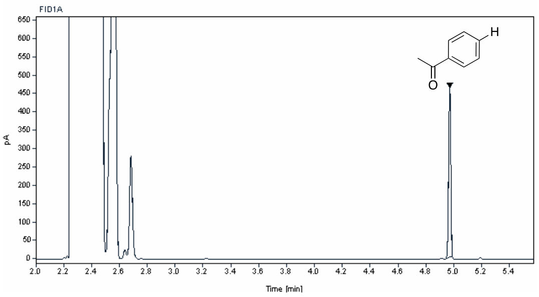


**Chromatogram after reaction**

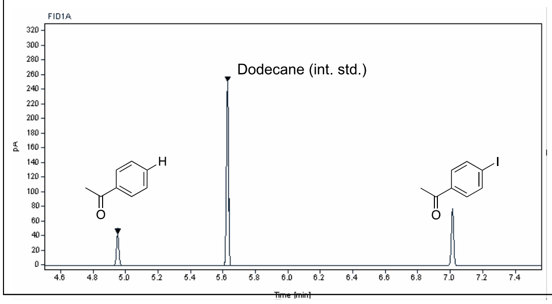

**Chromatogram of comercial reactant 2-bromoquinoline**

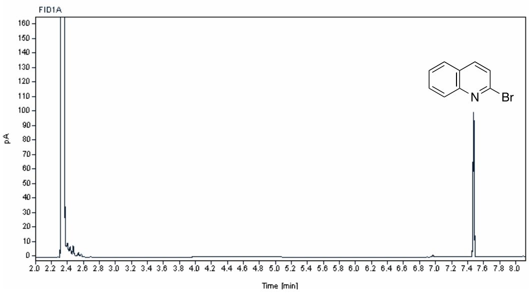


**Chromatogram of the commercial product quinoline**

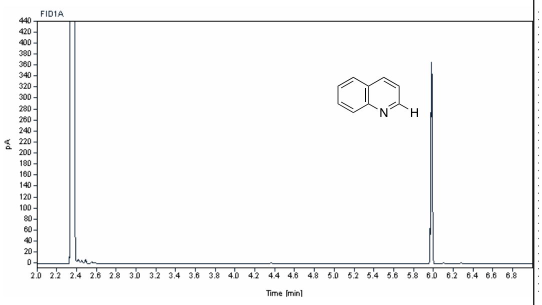


**Chromatogram after reaction**

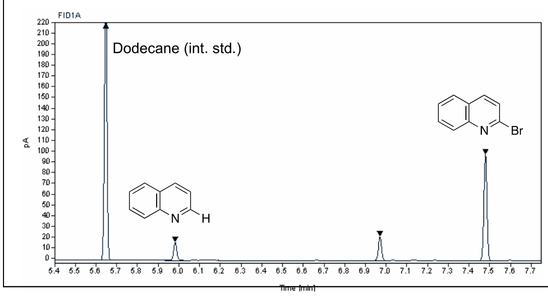


**Chromatogram of comercial reactant 3-bromoquinoline**

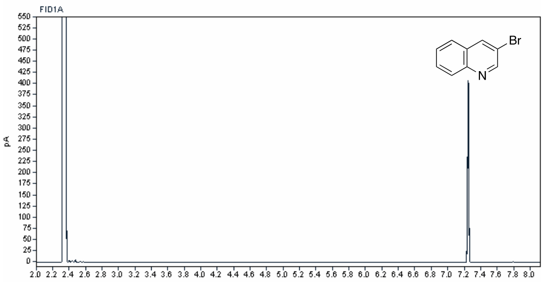


**Chromatogram of the commercial product quinoline**

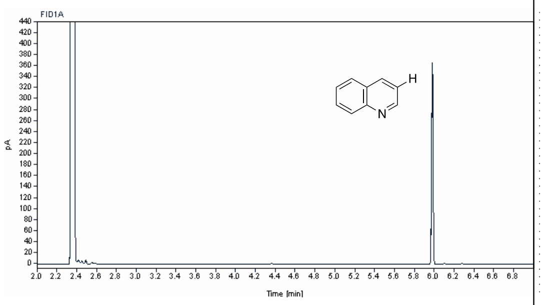


**Chromatogram after reaction**

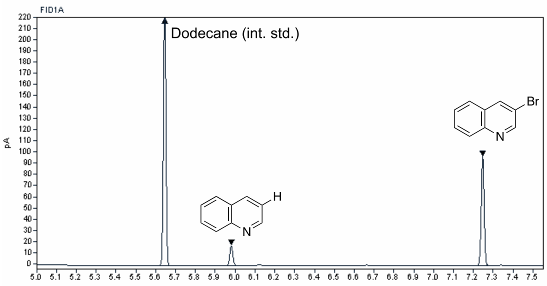

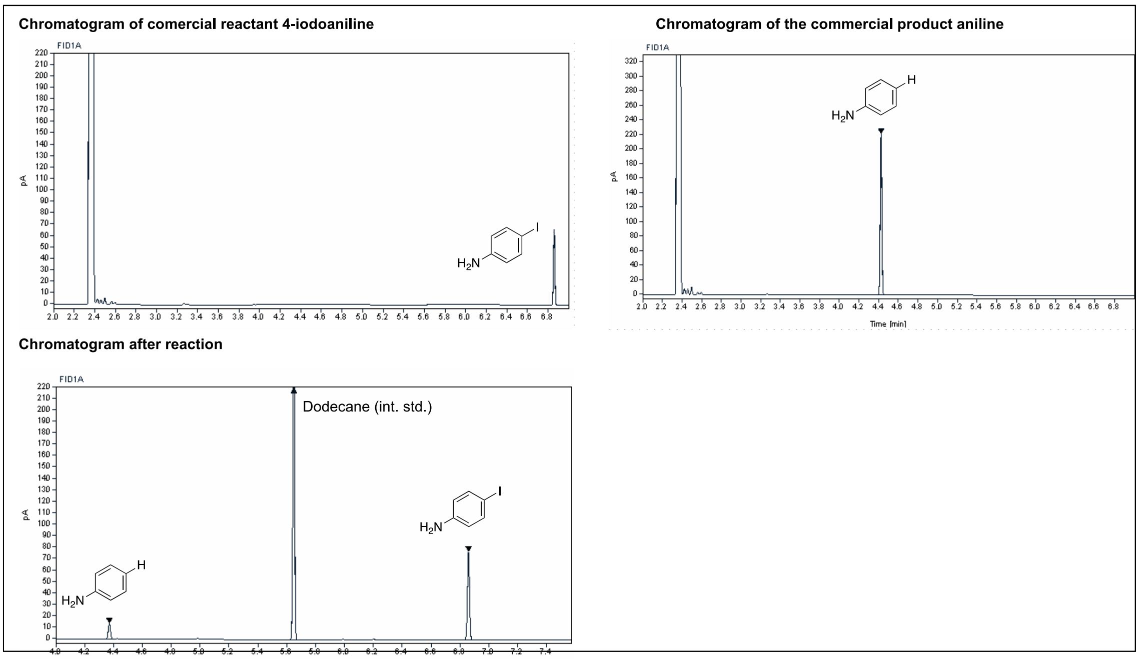


**Figure S3.** Representative GC-FID chromatograms of the photoreduction of different substates using laser pointers. The chromatograms of commercial reactants and products are also shown in each case as reference.

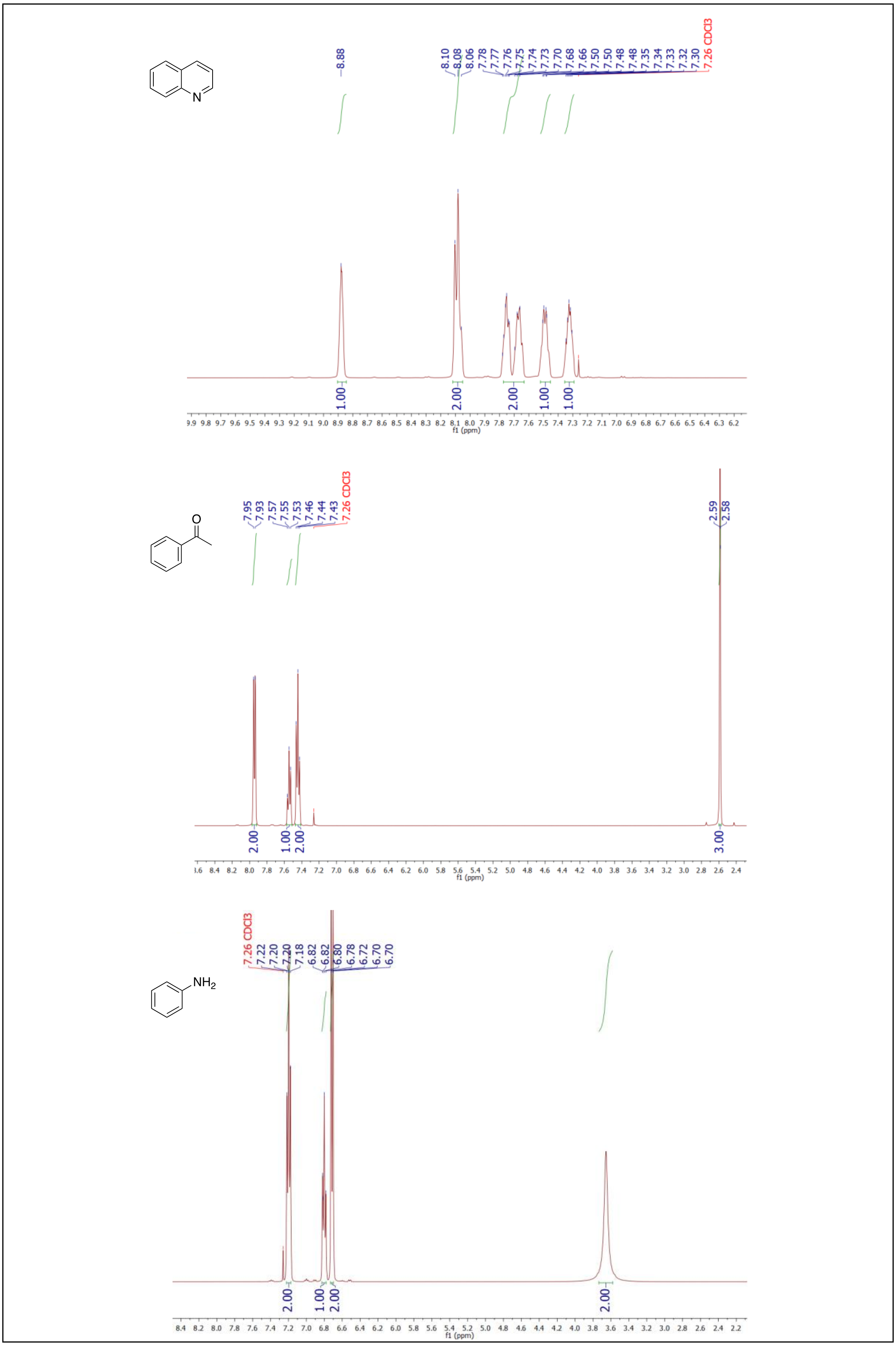


**Figure S4** Representative $^{1}H$ NMR spectra of reaction products.
